# Prolonged viral shedding and new mutations of COVID-19 could complicate the control of the pandemic

**DOI:** 10.1099/acmi.0.000133

**Published:** 2020-05-27

**Authors:** Chieh-Fan Chen, Tung-Yuan Tsai, Chen-Hui Yu, Hsiang-Lan Cheng, Ting-Yu Yeh

**Affiliations:** ^1^​ Department of Emergency, Kaohsiung Municipal United Hospital, Kaohsiung, Taiwan, ROC; ^2^​ Faculty of Medicine, Kaohsiung Medical University, Kaohsiung Medical University, Kaohsiung, Taiwan, ROC; ^3^​ Department of Internal Medicine, Kaohsiung Municipal United Hospital, Kaohsiung, Taiwan, ROC; ^4^​ Department of Infection Control, Kaohsiung Municipal United Hospital, Kaohsiung, Taiwan, ROC; ^5^​ Cheng Shiu University, Kaohsiung, Taiwan, ROC; ^6^​ Centers for Disease Control Ministry of Health and Welfare, The Executive Yuan, Kaohsiung, Taiwan, ROC; ^7^​ Agricultural Biotechnology Laboratory, Auxergen Inc. and GreenWave Bioscience, Columbus Center, Institute of Marine and Environmental Technology, University of Maryland, Baltimore, Maryland, USA

**Keywords:** COVID-19, coronavirus, RNA-dependent RNA polymerase, SARS-CoV-2, transmission dynamics

## Abstract

The studies of coronavirus disease 2019 (COVID-19) have mainly focused on epidemiological and clinical features of patients, but transmission dynamics of SARS-CoV-2 virus after patients have recovered is still poorly understood. Here we report a case with prolonged viral shedding of COVID-19 in Kaohsiung, Taiwan. This patient started to show myalgia and malaise in Wuhan, and the onset of the fever was on days 7–14 of the illness. All clinical and radiological results returned to normal after day 26, however, viral shedding was still evident 14 days later. Sequence analysis of the genome of the Taiwanese SARS-CoV-2 isolate from this patient reveals new mutations in viral replicase and ORF3a, indicating that COVID-19 evolves very quickly. Prolonged viral shedding and new mutations in the viral genome could potentially complicate the control of the COVID-19 pandemic.

## Introduction

A recent outbreak of pneumonia cases in Wuhan, China, was caused by a novel betacoronavirus, the 2019 novel coronavirus (COVID-19) [1]. The initiation of the COVID-19 outbreak showed the potential link to a large seafood and live animal market, suggesting animal-to-person spread. Later, multiple cases of person-to-person spread were subsequently reported in countries outside China, including 53 countries worldwide [2]. Some international destinations also have community spread currently. So far, the complete clinical picture and transmission dynamics with regard to COVID-19 are not fully understood. Viral shedding in various periods of the clinical course were observed in different biological samples, including nasopharyngeal and stool specimens. However, little has been studied to follow up the COVID-19 patients who have recovered from clinical symptoms. Lan *et al*. reported that after hospital discharge or discontinuation of quarantine, the reverse-transcriptase (RT)-PCR tests on patients’ throat swabs were repeated 5 to 13 days later and all were positive [3]. These results indicate that viral shedding from recovered patients may be the potential challenge and risk to quarantine COVID-19 patients in the hospital or at home.

On 31 December 2019, hospitals in Taiwan set up a patient flow plan to deal with potential cases and gathered data regarding patients’ travel, contact and exposure histories, in addition to fever or respiratory symptoms. Here we report the clinical course of a patient in Kaohsiung, Taiwan with prolonged viral shedding of COVID-19 for 40 days and novel viral sequence mutations.

## Case report

On 23 January 2020, a 59-year-old Taiwanese male with malaise and myalgia was referred by a local physician to our Emergency Department in Kaohsiung, Taiwan. We learned he had recently worked in Wuhan and placed him in quarantine in a negative pressure room within 7 min. This patient had reported an elevated body temperature (BT) of 37.5 °C and was prescribed antipyretic medications on 20 January 2020 in Wuhan. He returned to Kaohsiung by plane on January 21. His initial vital signs revealed BT of 37.3 °C, blood pressure of 173/104 mm Hg, pulse rate of 100 beats per min, respiratory rate of 16 breaths per min, and oxygen saturation of 100 % via room air. The patient does not have a history of chronic diseases. Lung auscultation revealed minimal crackle at posterior aspects of bilateral lower lung fields. Chest radiography presented as suspicious for increased infiltrations at bilateral lower lung fields, and the right lung is predominant (Fig. S1, available in the online version of this article). We reported this to the Taiwan Centers for Disease Control (CDC) as a case of severe pneumonia with novel pathogens. Throat swab and sputum specimens were sent to the CDC and were confirmed positive for COVID-19 using a real-time RT-PCR (rRT-PCR) assay performed the next day (Table 1). The patient became the second case of COVID-19 confirmed in Taiwan since then.

**Table 1.** Symptoms, maximal body temperature, RT-PCR results of viral RNA according to day of illness, 20 January to 28 February 2020

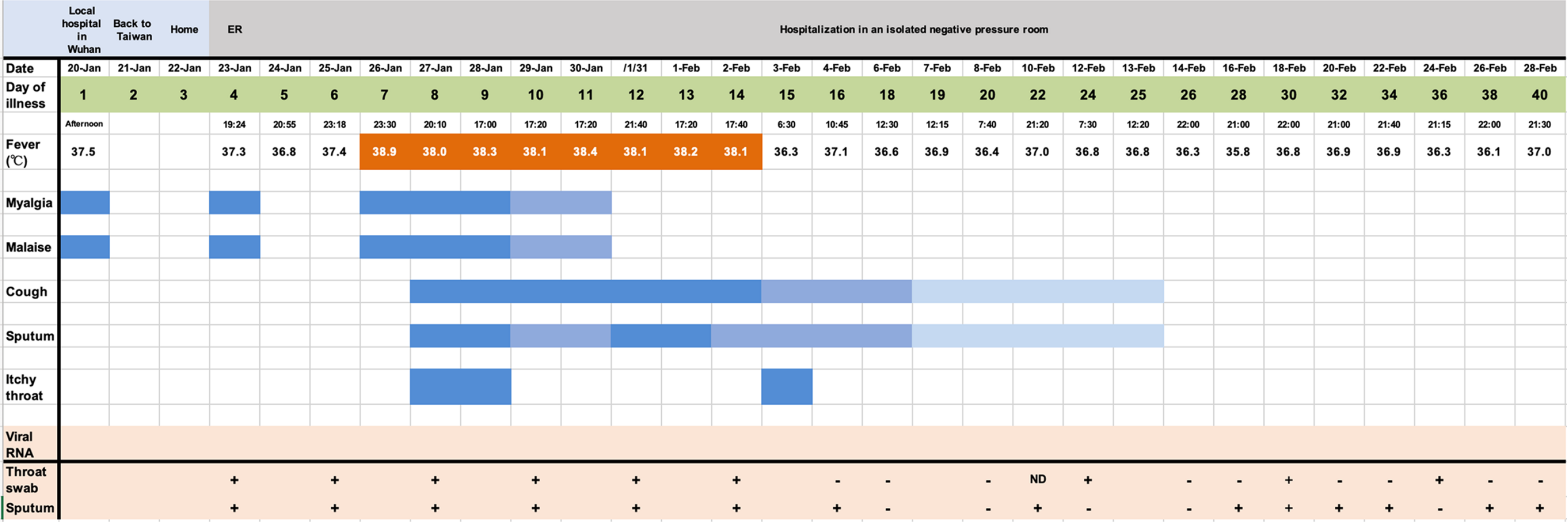

After admission, the patient’s white blood counts showed leucopenia (2.84×10^9^ l^−1^) on illness day 5 (Table 2) as reported [4, 5]. He received empiric antibiotic treatment with azithromycin (500 mg/day, illness day 4–8), then intravenous administration of ceftriaxone (1000 mg/12 h, illness day 8–16) and levofloxacin (750 mg/day, illness day 8–13) in association with supportive care. During the ensuing 6 days, he showed signs of mild fever, myalgia, malaise, and a peak fever (38.9 °C) on illness day 7. A more productive cough followed on the eighth day. He did not have nausea, vomiting, diarrhea or abdominal discomfort. Chest x-ray showed occasional infiltration with consolidation at bilateral lower lung field until illness day 23 (Figs S2–S10). The patient’s fever subsided after illness day 14. The late worsening of this illness to a high peak fever with pneumonia is consistent with recent reports [4, 5]. However, while rRT-PCR tests had been negative twice (on days 18 and 20 after illness) and clinical symptoms improved significantly, the virus could be detected again from day 22 (Table 1). The viral shedding was still evident after 14 days of recovering from respiratory symptoms (day 40). Currently the patient is still in hospital under observation. So far, no evidence of infection in family or the community has been found after the CDC followed up with the people he had contact with in Taiwan.

**Table 2.** Clinical laboratory results

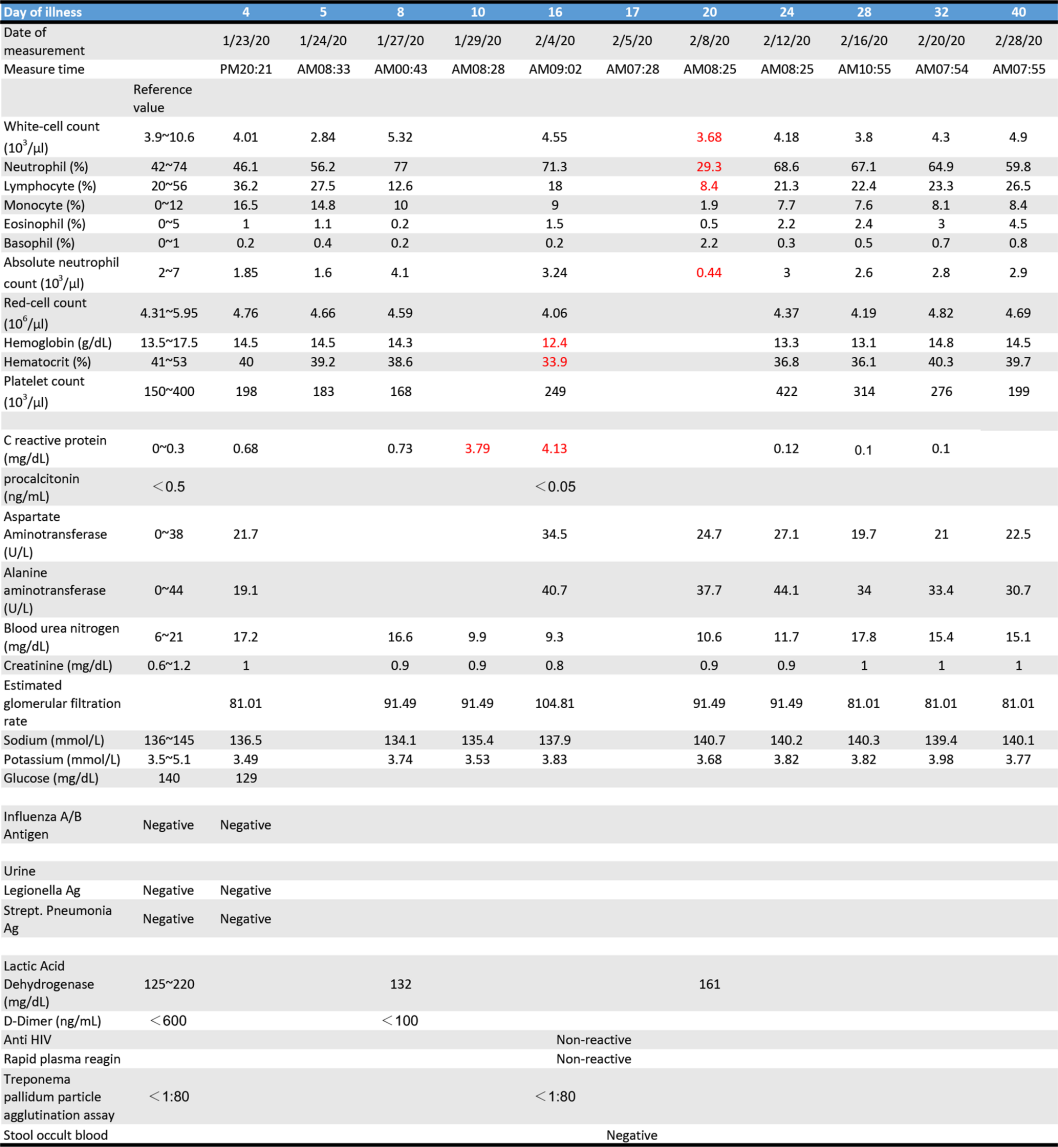

## Discussion

So far transmission dynamics of COVID-19 remain unclear. Current estimates of the incubation period of COVID-19 infection range from 1 to 14 days. However, certain case reports have suggested that the incubation period may reach 24 days [6], longer than the 14 days outlined in quarantine policies by the WHO and the US Centers for Disease Control and Prevention. The virus appears to be primarily transmitted through large droplets, but it has also been found in stool and blood samples. This raises the possibility about other potential modes of transmission. A potentially longer incubation period has critical implications for quarantine policies and prevention of spread. On the other hand, current criteria had to be met for hospital discharge or discontinuation of quarantine: (1) normal temperature lasting more than 3 days, (2) resolved respiratory symptoms, (3) substantially improved acute exudative lesions on chest computed tomography images, and (4) two consecutively negative RT-PCR test results separated by at least 1 day. Our patient met criteria for hospital discharge or discontinuation of quarantine in Taiwan (absence of clinical symptoms and radiological abnormalities and two negative rRT-PCR test results), but rRT-PCR results showed positive again even after all symptoms subsided after 14 days. If this is the characteristic transmission dynamics of COVID-19, it is bound to create a serious challenge.

**Fig. 1.**
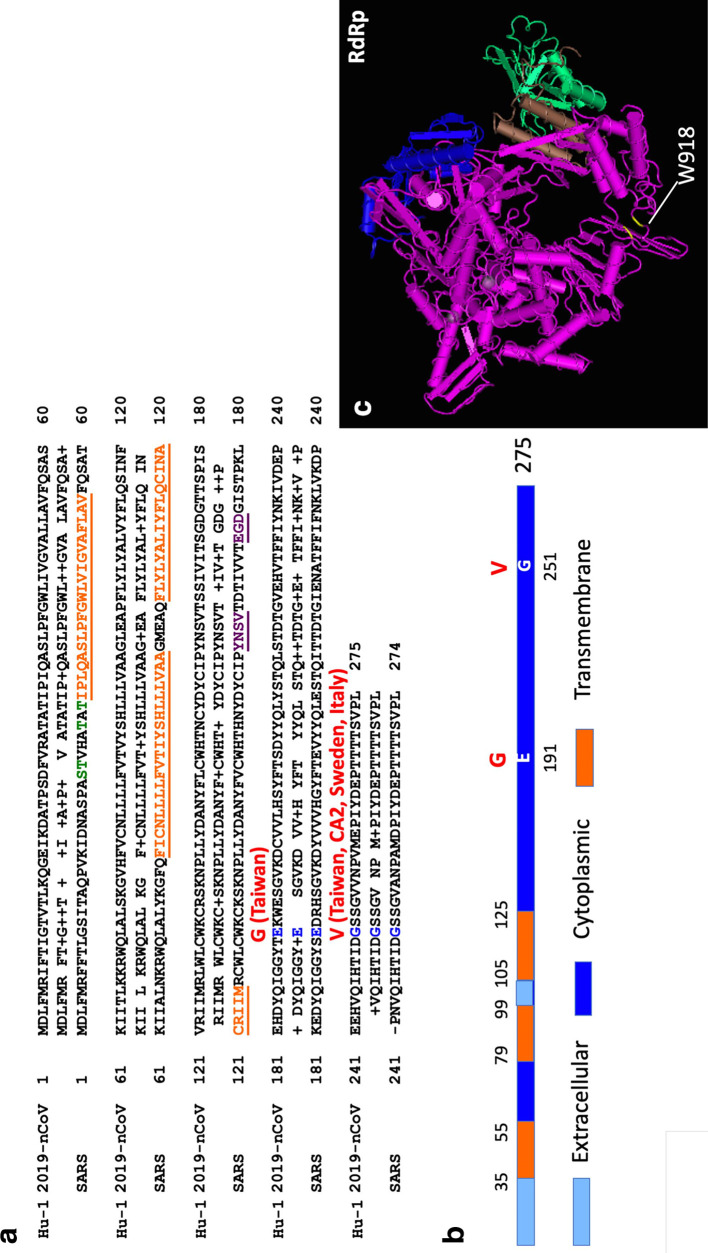
(a) Alignment of ORF3a protein of 2019-nCoV Hu-1 and SARS coronavirus. Transmembrane and Golgi localization motif are underlined in orange and purple, respectively. O-glycosylation sites are in green. E191G and G251V mutations (red) are shown in Taiwan and US-CA2, Sweden01, Italy-isl isolates. (b) Schematic representation of 2019-nCoV ORF3a protein. Both E191G and G251V mutations are in cytoplasmic region of Orf3a. (c) Mutation in tryptophan 913 (W913) is located in the thumb domain of RdRp. Structure of viral RNA-dependent RNA polymerase (RdRp) is based on SARS [10].

On 27 January, Taiwan CDC released the viral genome sequence from this patient (GSIAD; https://www.gisaid.org/) [7], which reveals novel features. Compared to all Wuhan isolates [8], the Taiwan isolate contains one synonymous substitution in nucleotide 26 141 (G>T), and two unique nonsynonymous substitutions in nucleotide 16 185 (G>T) and 25 961 (A>G), which generate the replacement of ORF1b tryptophan 913 (W913) with cysteine, and ORF3a glutamic acid 191 (E191) with glycine (G). Both ORF1b W913 and ORF3a E191 are conserved among SARS coronavirus, bat BtRf-BetaCoV, BtRs-BetaCoV and all other isolates of 2019-nCoV, but not Taiwan isolate (Fig. 1a). In addition, we found that Orf3a G251 of Wuhan isolates is replaced with valine in the Taiwan and the USA CA2, Sweden01, Italy-isl isolates. Both E191G and G251V mutations are located in the cytoplasmic regions of ORF3a protein (Fig. 1b). Mutations in ORF3a may play roles in attenuating interferon responses and innate immunity. Coronavirus spike and ORF3a proteins may show signs of positive selection of virus evolution [9]. In addition, ORF1b W913 is located in the thumb domain of NSP12/viral RNA-dependent RNA polymerase (replicase) (Fig. 1c), mutations in which lead to the emergence of drug-resistant virus variants [10–12]. Whether or not these mutations cause prolonged viral shedding of this patient is still unknown. As this virus continues to evolve, the influence of ORF1b and ORF3a mutations on the etiology and epidemic spread of COVID-19 warrants further investigation.

The viral evolution and COVID-19 outbreak are moving quickly. Our report provides a successful treatment strategy. Since mutations in viral replicase usually lead to drug-resistant virus variant, antiviral treatment should be evaluated more carefully. If prolonged viral shedding is commonly found in COVID-19 patients, the current protocol of hospital discharge or discontinuation of quarantine should be renewed as soon as possible.

## Supplementary Data

Supplementary material 1
