# Supplementary material for: Prolonged viral shedding and new mutations of COVID-19 could complicate the control of the pandemic
Source: Access Microbiol. 2020 May 27;2(7):acmi000133. doi: 10.1099/acmi.0.000133 (PMC7497832; doi:10.1099/acmi.0.000133)
Supplement: Supplementary material 1 [file acmi-2-133-s001.pdf]

Figure legend

Figure S1. Posteroanterior Chest Radiograph, January 23, 2020 (Illness Day 4). Emergency physician suspected that increased infiltrations at bilateral lower lung fields, right is predominant. Radiologist commended that increased infiltrations at right lower lung. Bronchopneumonia was suspected.

Figure S2. Posteroanterior Chest Radiograph, on January 24, 2020 (Illness Day 5). No significant change of increased infiltrations at right lower lung. Bronchopneumonia was suspected.

Figure S3. Posteroanterior Chest Radiograph, January 26, 2020 (Illness Day 7). Interval development of bronchopneumonia with increased infiltrations at left lower lung. Interval mild progression of bronchopneumonia at right lower lung.

Figure S4. Posteroanterior Chest Radiograph, January 31, 2020 (Illness Day 12). Interval progression of bronchopneumonia at both lungs with both lower lungs predominance.

Figure S5. Posteroanterior Chest Radiograph, February 1, 2020 (Illness Day 13). Persistent bronchopneumonia with increased infiltrations at both lungs with both lower lungs predominance.

Figure S6. Posteroanterior Chest Radiograph, February 3, 2020 (Illness Day 15). Bronchopneumonia with increased infiltrations at both lungs with both lower lungs predominance. Interval improvement at left lower lung.

Figure S7. Posteroanterior Chest Radiograph, February 5, 2020 (Illness Day 17). Interval progression of bronchopneumonia with increased infiltrations at both lungs with right lower lung predominance.

Figure S8. Posteroanterior Chest Radiograph, February 7, 2020 (Illness Day 19).

Figure S9. Posteroanterior Chest Radiographs, February 9, 2020 (Illness Day 21). Persistent bronchopneumonia with increased infiltrations at both lower lungs.

Figure S10. Posteroanterior Chest Radiograph, February 11, 2020 (Illness Day 23). Persistent bronchopneumonia with increased infiltrations at both lower lungs.

.

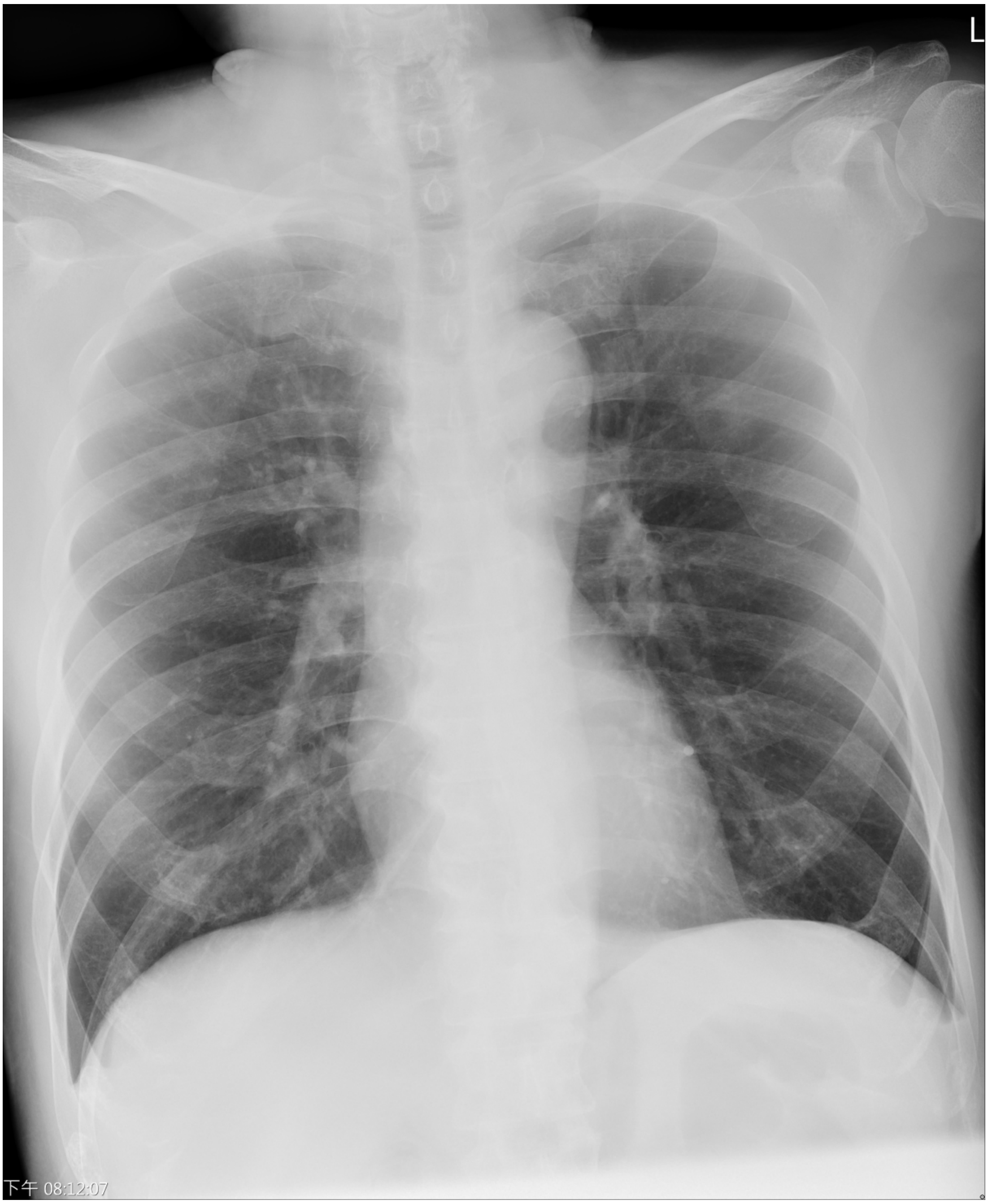

Figure S1. Posteroanterior Chest Radiograph, January 23, 2020 (Illness Day 4). Emergency physician suspected that increased infiltrations at bilateral lower lung fields, right is predominant. Radiologist commended that increased infiltrations at right lower lung. Suspect bronchopneumonia.

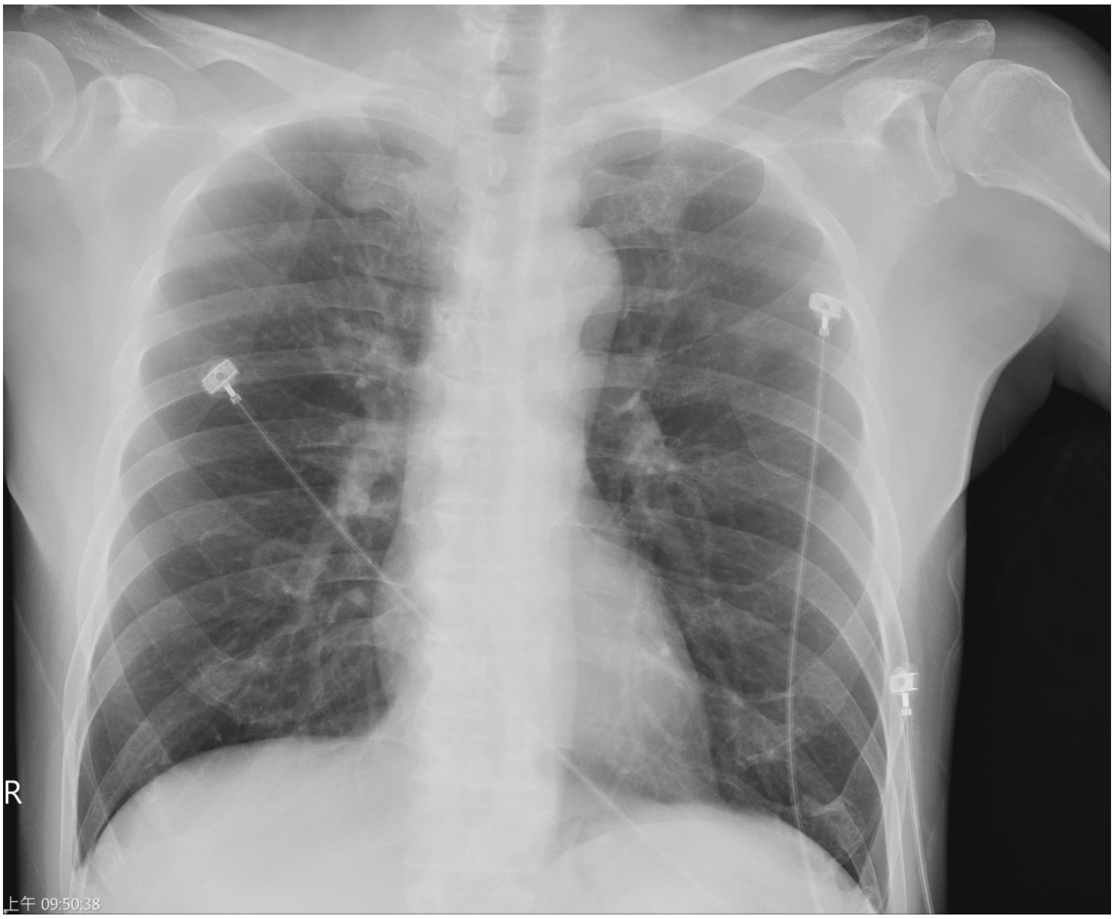

Figure S2. Posteroanterior Chest Radiograph on January 24, 2020 (Illness Day 5). No significant change of increased infiltrations at right lower lung. Bronchopneumonia was suspected.

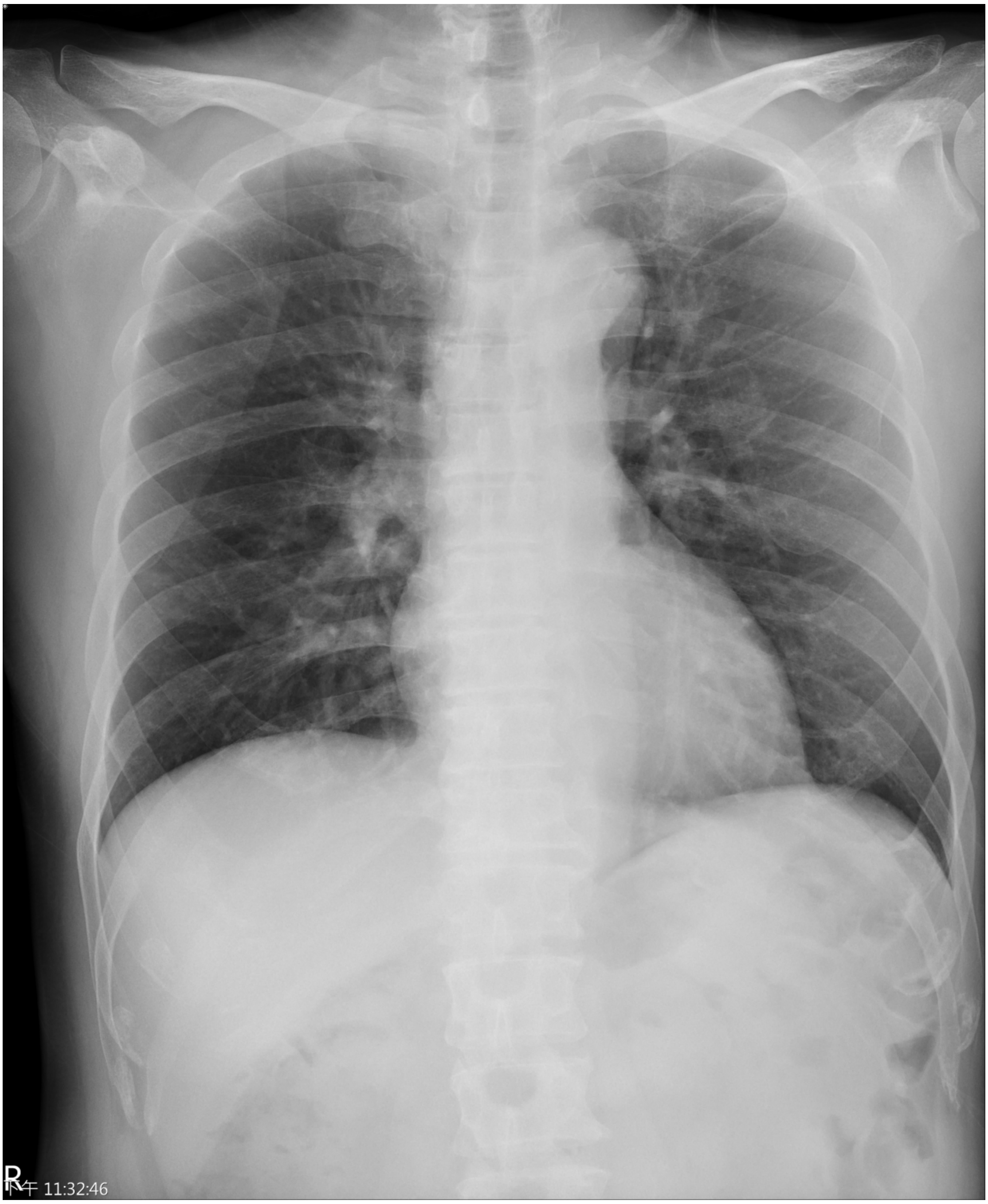

Figure S3. Posteroanterior Chest Radiograph, January 26, 2020 (Illness Day 7). Interval development of bronchopneumonia with increased infiltrations at left lower lung. Interval mild progression of bronchopneumonia at right lower lung.

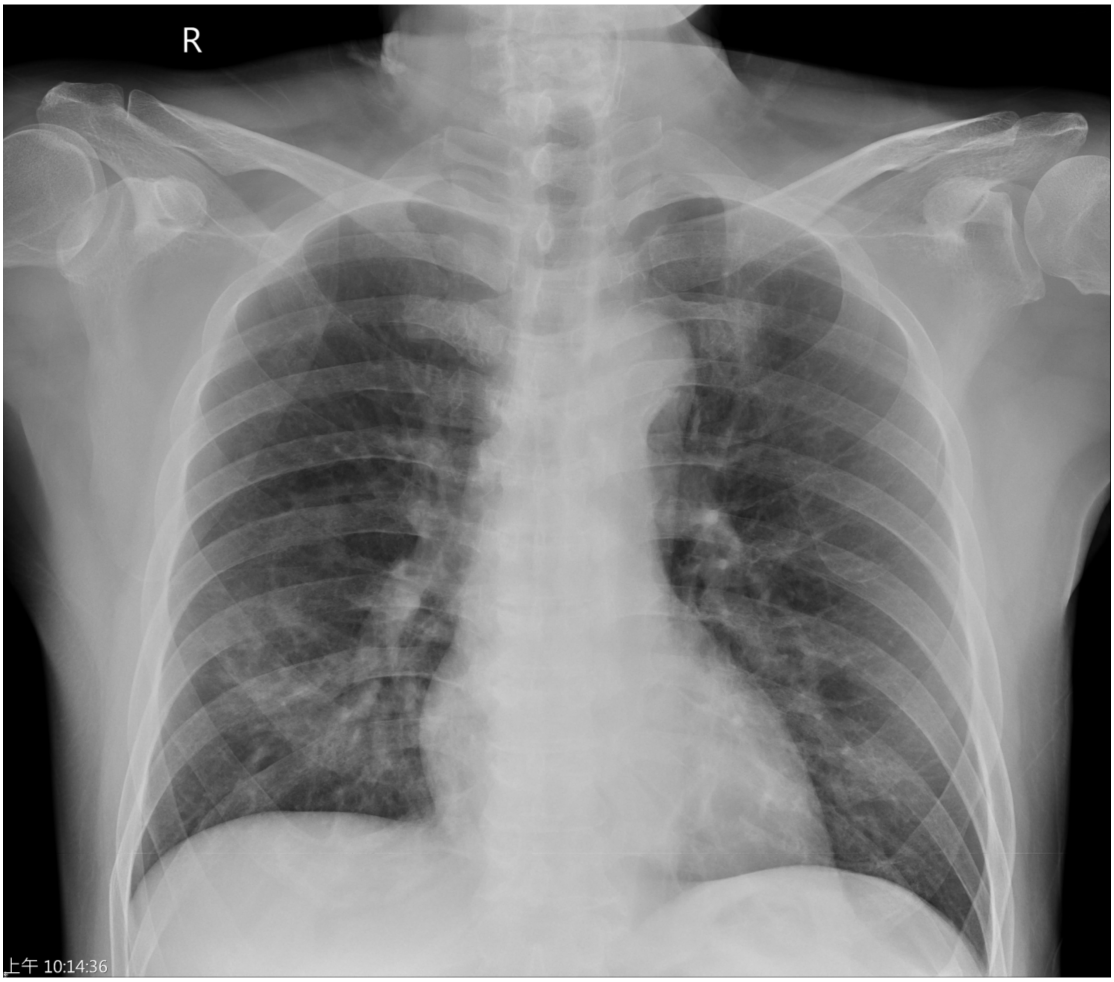

Figure S4. Posteroanterior Chest Radiograph, January 31, 2020 (Illness Day 12). Interval progression of bronchopneumonia at both lungs with both lower lungs predominance.

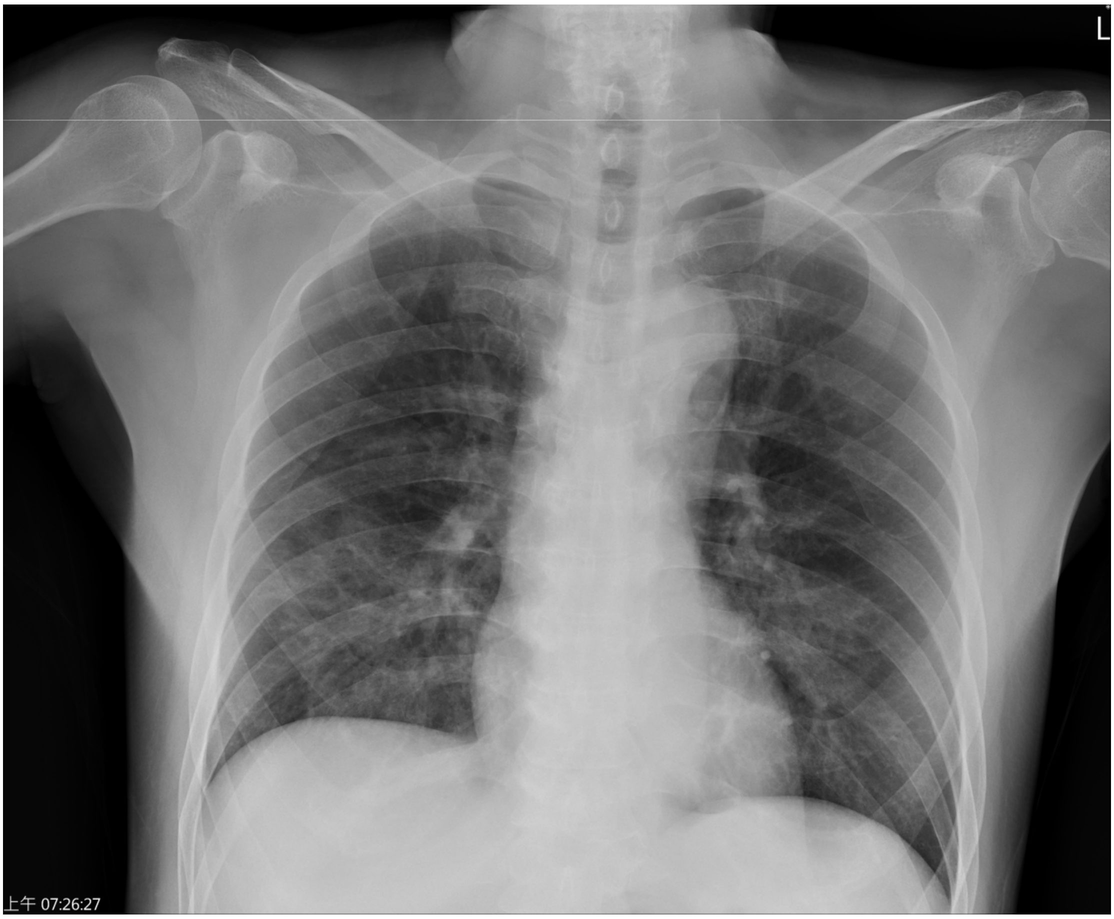

Figure S5. Posteroanterior Chest Radiograph, February 1, 2020 (Illness Day 13). Persistent bronchopneumonia with increased infiltrations at both lungs with both lower lungs predominance.

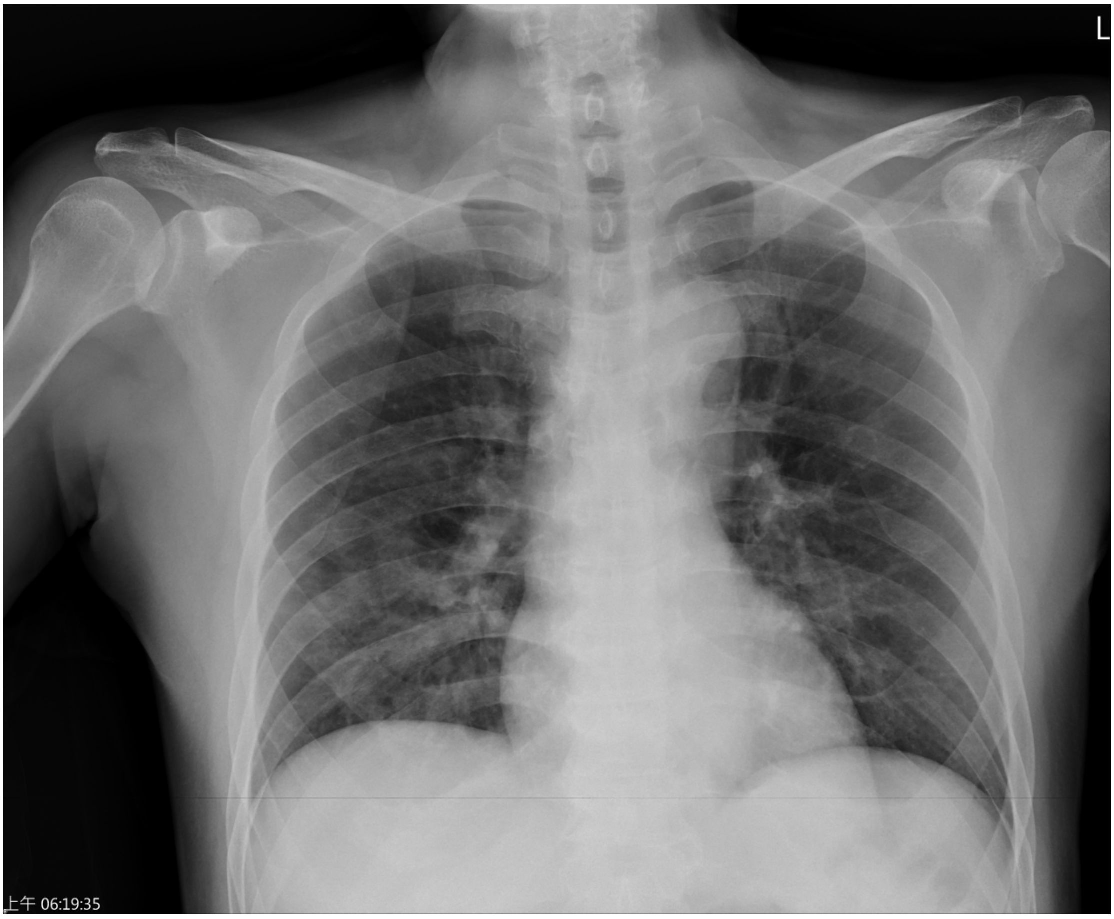

Figure S6. Posteroanterior Chest Radiograph, February 3, 2020 (Illness Day 15). Bronchopneumonia with increased infiltrations at both lungs with both lower lungs predominance. Interval improvement at left lower lung.

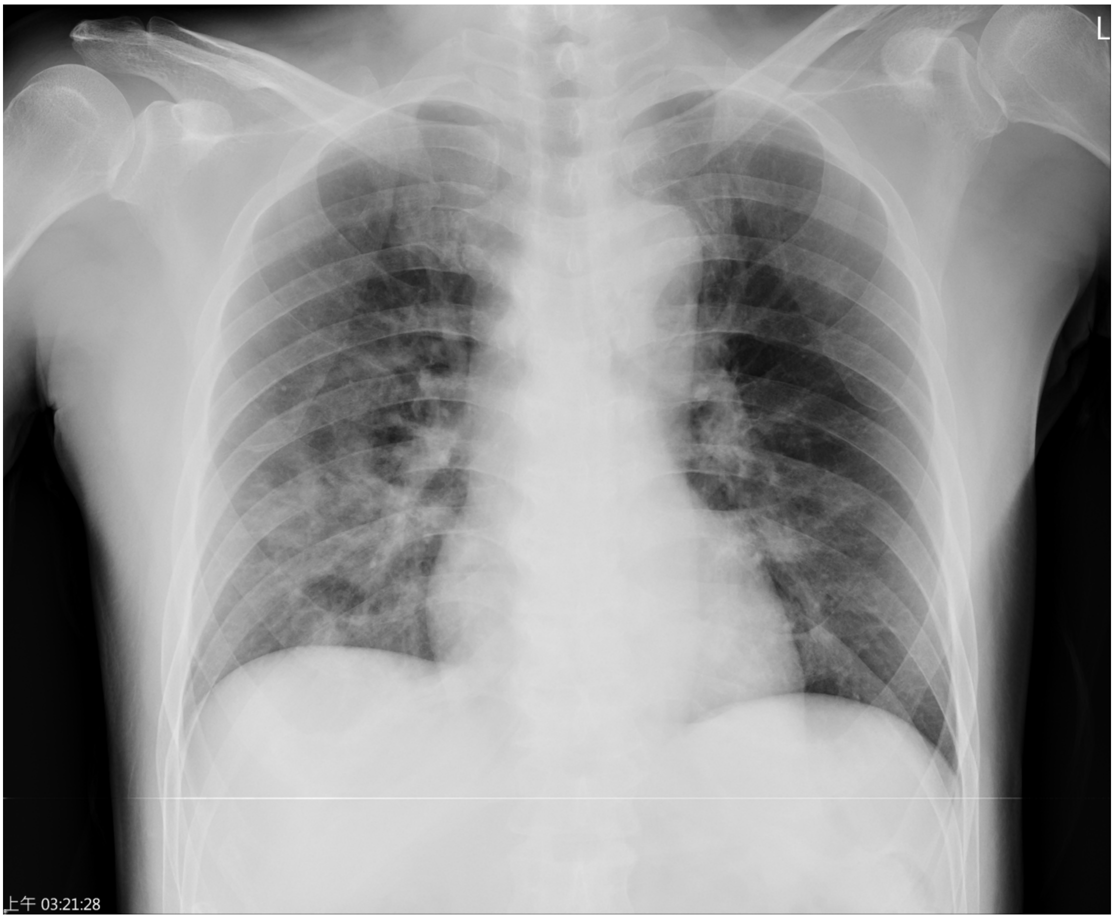

Figure S7. Posteroanterior Chest Radiograph, February 5, 2020 (Illness Day 17). Interval progression of bronchopneumonia with increased infiltrations at both lungs with right lower lung predominance.

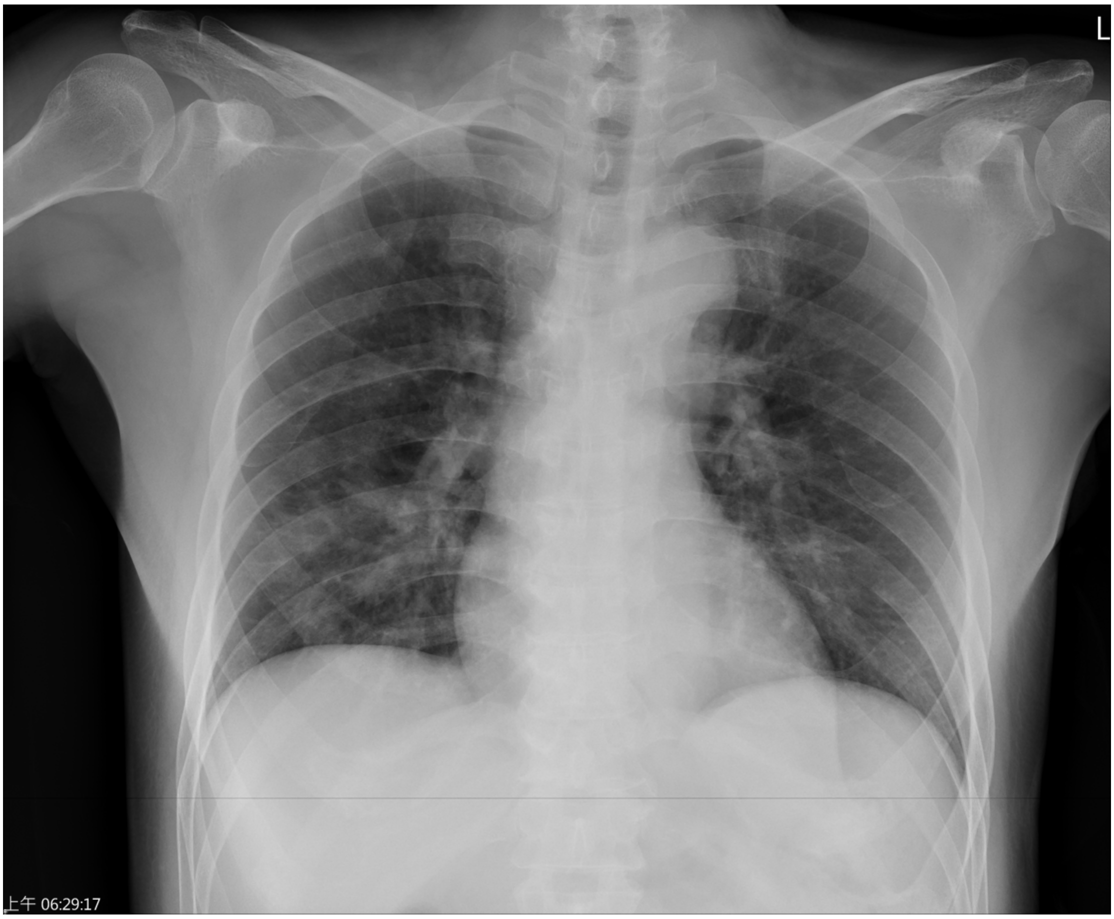

Figure S8. Posteroanterior Chest Radiograph, February 7, 2020 (Illness Day 19).

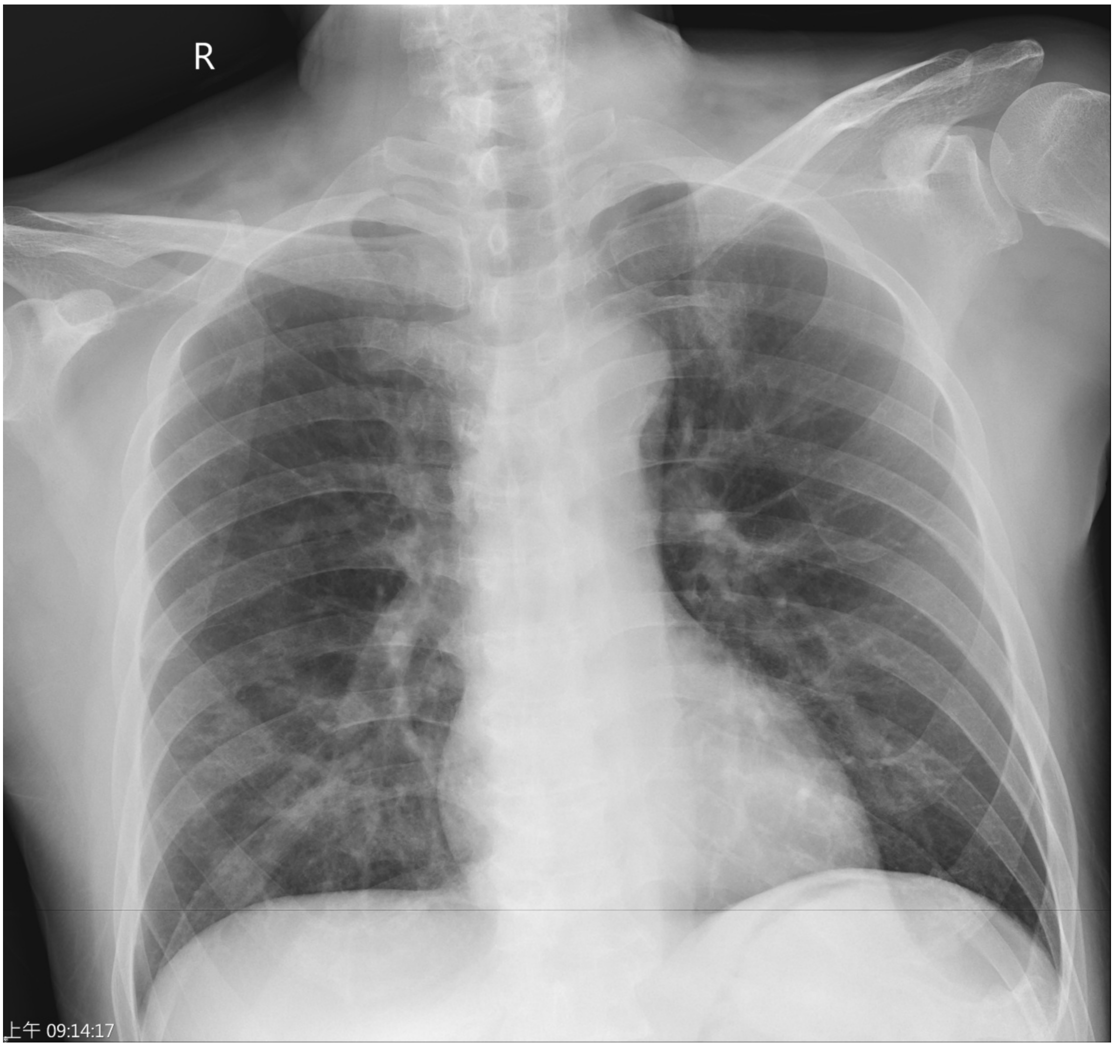

Figure S9. Posteroanterior Chest Radiograph, February 9, 2020 (Illness Day 21). Persistent bronchopneumonia with increased infiltrations at both lower lungs.

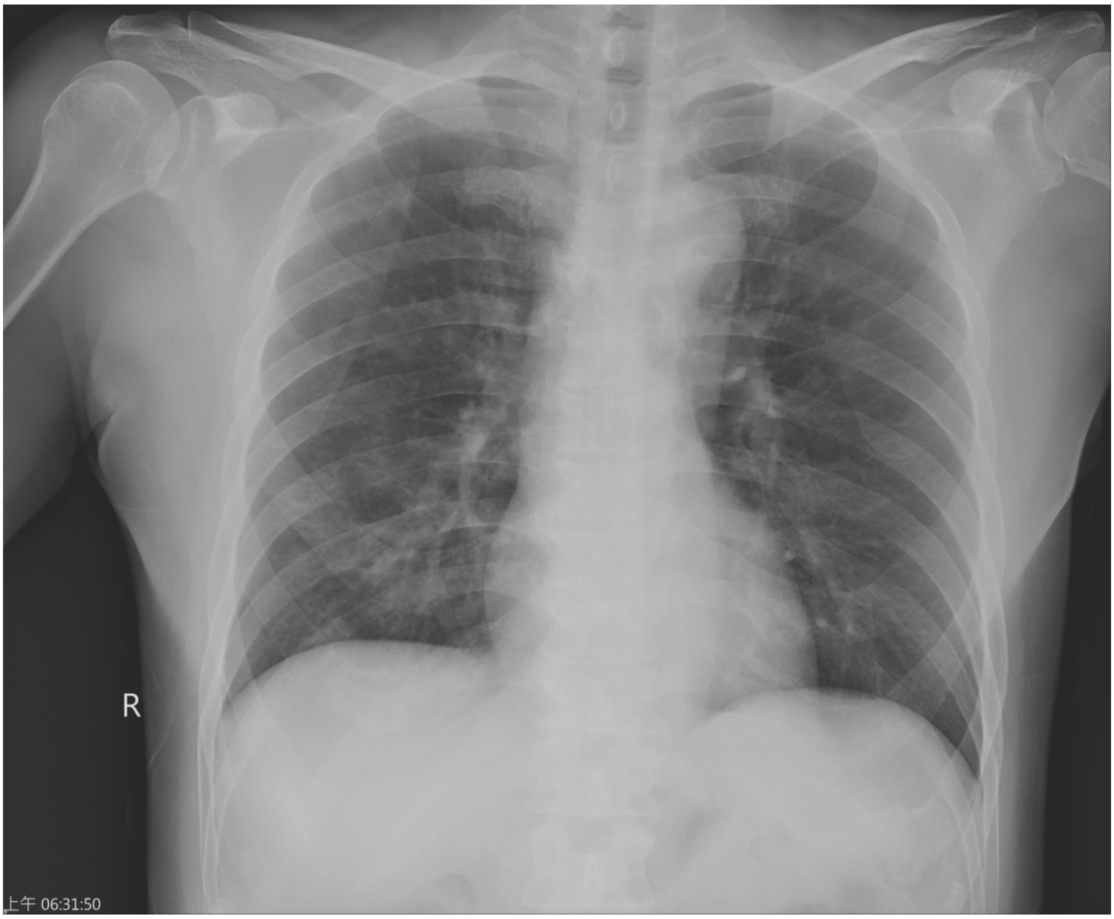

Figure S10. Posteroanterior Chest Radiograph, February 11, 2020 (Illness Day 23). Persistent bronchopneumonia with increased infiltrations at both lungs with lower lungs predominance.
